# SPRTN preserves RNA polymerase I transcription under genotoxic drug stress in lung adenocarcinoma

**DOI:** 10.3389/fonc.2026.1917106

**Published:** 2026-08-17

**Authors:** Mansi Sharma, Srikanth Karnati, Süleyman Ergün, Srivatsava Naidu

**Affiliations:** 1 Department of Biomedical Engineering, Indian Institute of Technology Ropar, Punjab, India; 2 Institute of Anatomy and Cell Biology, University of Würzburg, Würzburg, Germany

**Keywords:** chemoresistance, cisplatin, DNA-protein crosslinks, doxorubicin, genotoxic chemotherapy, lung adenocarcinoma, RNA polymerase I (Pol I), SPRTN

## Abstract

**Background:**

Hyperactivated RNA polymerase I (Pol I)-driven ribosomal DNA (rDNA) transcription sustains malignant growth and chemotherapy tolerance in lung adenocarcinoma (LUAD). Genotoxic chemotherapeutics such as doxorubicin and cisplatin preferentially damage rDNA by inducing DNA-protein crosslinks (DPCs), yet how tumor cells preserve Pol I transcriptional output under this damage remains poorly understood.

**Methods:**

We investigated SPRTN function in LUAD using complementary approaches spanning molecular, cellular, and *in vivo* levels, including DNA-protein crosslink isolation, chromatin immunoprecipitation, nascent RNA synthesis assays, apoptosis and invasion assays in SPRTN loss- and gain-of-function cell models, mouse xenograft studies, and transcriptomic analysis of clinical LUAD datasets.

**Results:**

SPRTN was enriched at the rDNA promoter, limited basal DPC accumulation, sustained Pol I transcriptional output, suppressed the p53-p21 nucleolar stress checkpoint, and promoted Pol I-dependent proliferation. Under doxorubicin or cisplatin treatment, SPRTN was recruited to G-quadruplex-rich rDNA promoters, a process associated with PARP-1 co-recruitment, and facilitated DPC resolution and transcriptional recovery. Catalytically inactive SPRTN failed to resolve rDNA-DPCs and restore Pol I output under both basal and drug-treated conditions. Doxorubicin- and cisplatin-resistant LUAD cells exhibited elevated SPRTN expression and enhanced rRNA synthesis relative to parental cells. SPRTN knockdown increased apoptotic response to doxorubicin and cisplatin, and co-inhibition of SPRTN and Pol I additively induced apoptosis in both parental and resistant cells. SPRTN further promoted Pol I-dependent migration and invasion. Transcriptomically, SPRTN was upregulated in LUAD, correlated with advanced disease stage and reduced overall survival, and was transcriptionally activated by ZEB1 and post-transcriptionally repressed by miR-195-5p. *In vivo*, SPRTN silencing reduced tumor burden and suppressed Pol I transcription, with further reduction upon doxorubicin treatment.

**Conclusion:**

These findings establish SPRTN as an rDNA-associated repair factor that couples genotoxic damage resolution to Pol I transcriptional resilience, defining the SPRTN-Pol I axis as a determinant of nucleolar homeostasis and therapeutic vulnerability in lung adenocarcinoma.

## 1 Introduction

RNA polymerase I (Pol I) transcribes nucleolar ribosomal DNA (rDNA) to produce ribosomal RNA (rRNA), the rate-limiting step in ribosome biogenesis, and its activity governs cell growth, proliferation, differentiation, and apoptosis (1). Pol I output requires the assembly of a dedicated pre-initiation complex (PIC) comprising SL1, UBF, RRN3, and Pol I at the rDNA promoter, and is stimulated by mitogenic pathways (Ras/MAPK, PI3K, and mTOR) and oncogenic regulators including c-MYC and NPM1, whereas tumor suppressors pRB and p53 restrain PIC formation (2, 3). Hyperactivation of Pol I transcription is a frequent feature of malignancy, associated with uncontrolled proliferation, epithelial-mesenchymal transition (EMT), poor prognosis, and resistance to therapy (4–6). In lung adenocarcinoma (LUAD), we previously identified a microRNA(miRNA)-circRNA axis that elevates Pol I transcription and showed that genetic inhibition of PIC components suppresses tumor growth and enhances cisplatin (Cis) response (7). We further demonstrated that LINC01116-mediated Pol I transcription activation promotes oncogenic phenotypes and reduces drug responsiveness in LUAD (8). Together, these findings underscore that intricate regulatory circuits sustain Pol I activity in cancer and highlight its indispensable role in maintaining malignant phenotypes and genotoxic drug tolerance. Consequently, Pol I hyperactivity represents a tractable therapeutic vulnerability, and selective inhibitors such as CX-5461 (9), BMH-21 (10), and NSH76 (11) have been developed to target the Pol I transcriptional machinery.

The rDNA locus is GC-rich and structurally complex, forming R-loops and G-quadruplexes (G4s) during high Pol I transcriptional activity (12, 13). These non-canonical structures impede transcriptional elongation and promote the formation of DNA-protein crosslinks (DPCs) involving topoisomerases and Pol I-associated factors. Unresolved DPCs arrest Pol I transcription, disrupt nucleolar architecture, and activate p53-mediated cell cycle arrest and apoptosis, collectively termed nucleolar stress (12). Doxorubicin (Dox) and Cis preferentially damage rDNA by stabilizing G4 structures, inducing DPCs, and triggering nucleolar stress, mechanisms that underlie a significant component of their cytotoxic action (14–18). Despite this, how cancer cells maintain rDNA transcriptional integrity under the combined burden of high rRNA demand and genotoxic insult, and whether dedicated repair mechanisms at rDNA contribute to tumor survival and drug adaptation, remain poorly understood.

SPRTN is a DNA-dependent metalloprotease essential for replication-coupled DPC removal, genome stability, and prevention of protein-DNA adduct accumulation and replication fork collapse (19–21). While SPRTN function has been extensively characterized in the context of DNA replication, its potential role in transcription-coupled DPC repair, particularly at the highly transcribed rDNA locus, has not been investigated. Given that rDNA sustains exceptionally high transcriptional flux and accumulates DPCs under both basal conditions and genotoxic stress, we hypothesized that SPRTN associates with rDNA, limits DPC burden, and thereby preserves Pol I transcriptional output under basal and drug-induced stress conditions.

Here, we show that SPRTN is enriched at rDNA promoter regions, limits basal DPC accumulation, sustains Pol I transcription, dampens the p53-p21 nucleolar-stress checkpoint, and promotes proliferation in LUAD cells. Under Dox or Cis treatment, SPRTN is recruited to G4-rich rDNA promoters in association with PARP-1, facilitating DPC resolution and enabling transcriptional recovery. Dox- and Cis-resistant LUAD cells adaptively upregulate SPRTN and rRNA synthesis; SPRTN knockdown increased apoptotic response to Dox and Cis, and co-inhibition of SPRTN and Pol I additively induces apoptosis in both parental and resistant cells. SPRTN further supports Pol I transcription-linked migration, invasion, and mesenchymal gene expression. Transcriptomically, SPRTN is upregulated in LUAD and regulated by ZEB1 and miR-195-5p. *In vivo*, SPRTN knockdown suppresses Pol I transcription and tumor growth, with further reduction in tumor burden upon Dox treatment. Collectively, these findings establish SPRTN as a nucleolar stress-responsive factor that maintains rDNA transcriptional integrity and promotes genotoxic drug adaptation, highlighting the SPRTN-Pol I axis as a therapeutic vulnerability in LUAD.

## 2 Materials and methods

Primer sequences, plasmids, and antibodies used in this study are listed in **Supplementary Table 1**. G4-rich rDNA core promoter and G4-depleted 5′ ETS primer sets were designed based on the G-quadruplex-forming sequence motifs within the human rDNA repeat (12, 13).

### 2.1 In silico analysis

SPRTN expression in LUAD was queried from The Cancer Genome Atlas (TCGA) using GEPIA (gepia.cancer-pku.cn). Stagewise expression and survival analyses were performed with UALCAN (ualcan.path.uab.edu) and cBioPortal (cbioportal.org), respectively. Single-cell RNA sequencing (scRNA-seq) LUAD datasets were processed in the Loupe Browser to generate Uniform Manifold Approximation and Projection (UMAP) and k-means (k=6) clusters (10xgenomics.com). Gene ontology (GO) enrichment of SPRTN-high clusters was performed using iDEP.96 (bioinformatics.sdstate.edu/idep96). SPRTN-ZEB1 correlation analysis was performed via ONCODB (oncodb.org). Candidate miRNAs targeting the SPRTN 3′UTR were predicted using miRWalk (mirwalk.umm.uni-heidelberg.de), and the SPRTN-miR-195-5p expression correlation was evaluated using StarBase v2.0 (starbase.sysu.edu.cn).

### 2.2 Cell culture

A549, H23, and HEK293T cells were obtained from the National Centre for Cell Science, Pune, India, and authenticated at the source. A549-derived doxorubicin-resistant (A549-DoxR^+^) and cisplatin-resistant (A549-CisR^+^) cell lines were generated as previously described (8). A549, H23, A549-DoxR^+^ and A549-CisR^+^ cells were cultured in RPMI-1640 medium (Gibco, Waltham, MA, USA), and continuously maintained in 1 μM Dox (Sigma-Aldrich, St. Louis, MO, USA) or 1 μM Cis (Sigma-Aldrich, St. Louis, MO, USA), respectively. Prior to assays, resistant cells were cultured in drug-free medium for 48 h to minimize acute drug effects; untreated conditions refer to cells following this withdrawal period. HEK293T cells were cultured in DMEM (Gibco), supplemented with 10% heat-inactivated fetal bovine serum (FBS) (Gibco) and 100 U/mL penicillin-streptomycin (Gibco). Cells were maintained at 37 °C in a humidified incubator with 5% CO_2_, and regularly monitored for mycoplasma contamination.

### 2.3 Stable or transient transfections

Transfections were performed in 6-well plates with Lipofectamine 2000 (Invitrogen, Waltham, MA, USA) according to the manufacturer’s instructions. For stable knockdown, A549 cells were transfected with SPRTN shRNA vector (Merck, #TRCN0000073309, Darmstadt, Germany) or pLKO.1-puro control vector and selected with puromycin (0.5 µg/mL; Merck P8833). For stable overexpression, cells were transfected with an SPRTN full-length cDNA vector (Addgene, #110214) or pcDNA3 control vector and selected with Zeocin (50 µg/mL, InvivoGen, San Diego, CA, USA). For transient assays, A549 and H23 cells were transfected with 1 µg each of SPRTN shRNA vector or control vector; or SPRTN full-length cDNA or control vector. A549 cells were transfected with a ZEB1 expression vector or PARP-1 shRNA (Merck, #TRCN0000007929) alongside matched control vectors. For ZEB1 knockdown, A549 cells were transiently transfected with ZEB1-targeting small interfering RNA (siRNA) (30 nM; # sc-38643, Santa Cruz Biotechnology, Inc., TX, USA) or non-targeting scrambled siRNA control using Lipofectamine 2000 according to the manufacturer’s instructions. Cells were harvested 48 h post-transfection for RNA extraction and qPCR analysis. For miRNA experiments, cells were transfected with 50 nM scrambled control mimic or miR-195-5p mimic (RealGene, Gottingen, Germany). For catalytic inactive rescue experiments, A549 cells were transiently transfected with a catalytically inactive SPRTN mutant vector SPRTN-CD (kind gift from Dr. Swagata Halder, Plaksha University, Punjab, India) or wild-type SPRTN expression vector or empty vector control using Lipofectamine 2000 according to the manufacturer’s instructions. For rescue experiments, SPRTN-CD-expressing cells were subsequently transfected with wild-type SPRTN expression vector or empty vector control 24 h after initial transfection. For Pol I-specific inhibition, A549 cells stably expressing SPRTN shRNA or SPRTN overexpression vector were transiently transfected with TAF1C shRNA vector 1 µg (Sigma Aldrich) or non-targeting scrambled shRNA control vector (Sigma Aldrich) using Lipofectamine 2000 according to the manufacturer’s instructions.

### 2.4 Immunofluorescence microscopy

A549 cells (1 × 10³) cultured on coverslips were treated for 24 h with Dox (1 µM), Cis (1 µM), or DMSO. Cells were fixed with 4% paraformaldehyde (Sigma-Aldrich), permeabilized with 0.1% Triton X-100 (Sigma-Aldrich), and incubated overnight at 4 °C with anti-SPRTN (1:1000), anti-TAF1B (1:1000), or anti-NPM-1 (1:1000) primary antibodies. Alexa Fluor 488-conjugated anti-mouse (1:10000) and Texas Red-conjugated anti-rabbit (1:10000) secondary antibodies were applied for 1 h at room temperature (RT). Nuclei were counterstained with Hoechst 33342 (1:4000, 5 min; Invitrogen). Images were acquired on a Leica DMi8 fluorescence microscope (Leica Microsystems, Wetzlar, Germany) and fluorescence intensities were quantified on a per-field basis using LAS X software (Leica Microsystems). Fields with comparable cell density were selected for analysis. Mean fluorescence intensity per field was measured, and background correction was performed by subtracting the mean intensity of cell-free regions within the same field. Fluorescence values were normalized to the mean intensity of the control condition within each independent experiment.

### 2.5 RNA isolation and quantitative PCR

Total RNA from cultured cells and xenograft tissues was extracted using TRIzol reagent (Ambion, Thermo Fisher Scientific, Waltham, MA, USA) and quantified on a NanoDrop spectrophotometer (Thermo Fisher Scientific). For mRNA quantification, 1 µg of RNA was reverse transcribed using the PrimeScript RT Reagent Kit (Takara, Shiga, Japan). For miRNA quantification, 1 µg of RNA was reverse transcribed using universal and miRNA-specific primers. Quantitative PCR (qPCR) was performed using TB Green^®^ Premix Ex Taq™ (Tli RNaseH Plus; Takara) with gene-specific primers. γ-Actin and U6 small nuclear RNA served as internal controls for mRNA and miRNA quantification, respectively. Relative expression levels were calculated using the 2^^−ΔΔCt^ method.

### 2.6 Isolation of DNA-protein complexes

A549 cells (6 × 10^5^ per well) stably expressing SPRTN shRNA or SPRTN overexpression vector, and H23 cells (6 × 10^5^ per well) transiently expressing SPRTN shRNA or SPRTN overexpression vector, were treated with Dox (1 µM), Cis (1 µM), or DMSO for 24 h. For time-course analysis of DPC resolution, A549 empty vector, SPRTN shRNA, and SPRTN-overexpressing cells were treated with Dox (1 µM) or DMSO for 24 h, washed three times with PBS, and collected at 0, 6, 12, 18, and 24 h post-washout. For catalytic inactive rescue experiments, A549 cells transiently expressing empty vector, wild-type SPRTN, SPRTN-CD, or SPRTN-CD rescued with wild-type SPRTN, treated with Dox (1 µM) or DMSO for 24 h. DPCs were isolated using the Rapid Approach to DNA Adduct Recovery (RADAR) method (22). Briefly, cells were lysed in potassium dodecyl sulfate buffer, and DPC-bound and free DNA fractions were precipitated with 70% ethanol. Pellets were resuspended in 8 mM NaOH and quantified by NanoDrop spectrophotometry. For dot-blot analysis, equal amounts of DNA (300 ng per spot in 2 µL, normalized by NanoDrop quantification) were applied to methanol-activated PVDF membranes, air-dried (20 min), baked at 80 °C (2 h), and blocked with 5% BSA. Membranes were incubated overnight at 4 °C with anti-Topoisomerase IIα (Topo2A; 1:1000), followed by HRP-conjugated anti-rabbit secondary antibody (1:10000). Signals were visualized using enhanced chemiluminescence (ECL; Bio-Rad, Hercules, CA, USA) and quantified with Image Lab software (Bio-Rad). For RADAR-qPCR, DPC-bound and free DNA fractions were treated with proteinase K (Invitrogen) and RNase A (HiMedia, Maharashtra, India) prior to amplification with rDNA core promoter primers.

### 2.7 Chromatin immunoprecipitation assay

A549 cells stably expressing SPRTN shRNA or control vector, and A549-DoxR^+^ or A549-CisR^+^ cells (1 × 10^7^ per dish) were treated for 24 h with Dox (1 µM), Cis (1 µM), or DMSO. Cells were crosslinked with 1% paraformaldehyde (Sigma-Aldrich), quenched with 0.125 M glycine, lysed, and sonicated (32% amplitude, 15 cycles of 30 s ON/30 s OFF; SinapTec Lab 120) to generate 200–500 bp chromatin fragments. Chromatin was immunoprecipitated using Protein A magnetic beads (BioBharati, Kolkata, India) pre-incubated with anti-SPRTN, anti-PARP-1, or anti-ZEB1 antibodies, or IgG isotype controls (1:1000), for 3 h at RT. Immune complexes were eluted in 1% SDS 0.1/M NaHCO_3_, treated with RNase A and proteinase K, and purified DNA was analyzed by qPCR using gene-specific primers.

### 2.8 Apoptosis assay

Cells (1.5 × 10^5^ per well) were seeded in 6-well plates and treated for 24 h with DMSO, Dox (1 µM), Cis (1 µM), or BMH-21 (3 µM), alone or in combination. Apoptosis was assessed using the Annexin V-Propidium Iodide Apoptosis Detection Kit I (BD Biosciences, Franklin Lakes, NJ, USA) and analyzed on a BD Accuri™ C6 Plus flow cytometer (BD Biosciences). Data were quantified using FlowJo software (BD Biosciences). Cells were first identified based on forward scatter (FSC) and side scatter (SSC) characteristics to exclude debris. Cell doublets and aggregates were removed by sequential gating using FSC-A versus FSC-H. The resulting single-cell population was analyzed for Annexin V and propidium iodide (PI) fluorescence. Quadrant gates were established using unstained controls.

### 2.9 Cell proliferation assay

A549 cells (1 × 10³ per well) stably expressing SPRTN shRNA or SPRTN overexpression vector, and, A549 and H23 cells transiently expressing SPRTN shRNA or SPRTN overexpression vector, were seeded in 96-well plates and cultured overnight. Cells were then treated with BMH-21 (3 µM; Sigma-Aldrich) or DMSO. For Pol I-specific genetic inhibition, A549 cells or A549 cells stably expressing SPRTN shRNA or SPRTN overexpression vector were transiently transfected with TAF1C shRNA vector or non-targeting scrambled shRNA control. After 24 h, cell viability was assessed using Alamar Blue reagent (Invitrogen) per the manufacturer’s instructions. Absorbance was measured at 570 nm on a CLARIOstar microplate reader (BMG Labtech, Ortenberg, Germany).

### 2.10 5-Ethynyl uridine incorporation assay

Cells (1 × 10^5^ per well, 6-well plates) were seeded and treated with Dox (1 µM), Cis (1 µM), or DMSO for 24 h. Nascent RNA synthesis was assessed by pulsing cultures with 5-ethynyl uridine (EU; 100 µM; Sigma-Aldrich) for 1 h at 37 °C, 5% CO_2_, followed by processing using the Click-iT RNA Alexa Fluor Imaging Kit (Sigma-Aldrich). Fluorescence was imaged on a Leica DMi8 microscope (Leica Microsystems) and quantified on a per-field basis using LAS X software (Leica Microsystems). Five randomly selected non-overlapping fields per coverslip were imaged using identical acquisition settings. Quantification was performed on all acquired fields.

### 2.11 Immunoblotting

A549 cells (1.5 × 10^5^ per well, 6-well plates) stably expressing SPRTN shRNA or SPRTN overexpression vector were treated with BMH-21 (3 µM) or DMSO for 24 h. Total protein was extracted as previously described (23), quantified by Bradford assay (Bio-Rad), resolved by 8–10% SDS-PAGE, and transferred to PVDF membranes (Bio-Rad). Membranes were blocked with 5% BSA and incubated overnight at 4 °C with anti-SPRTN (1:1000), anti-p53 (1:1000), anti-β-actin (1:1000), or anti-GAPDH (1:1000) primary antibodies, followed by HRP-conjugated anti-rabbit secondary antibody (1:10000, 1 h, RT). Signals were detected by ECL (Bio-Rad) on a ChemiDoc imaging system (Bio-Rad, Puchheim, Germany) and quantified using Image Lab software. β-actin or GAPDH served as loading controls.

### 2.12 Luciferase reporter assay

The luciferase reporter assay was performed as previously described (23) using the Dual-Luciferase^®^ Reporter Assay System (Promega, Madison, WI, USA). Briefly, HEK293T cells (1 × 10^5^ per well, 12-well plates) were transfected with pGL3 constructs containing the SPRTN 3′UTR (Biotechdesk, Hyderabad, India) together with miR-195-5p mimics or non-targeting control mimics. After 24 h, cells were lysed in passive lysis buffer, and Firefly and Renilla luciferase activities were measured sequentially on a GloMax Navigator luminometer (Promega). Results are expressed as Firefly/Renilla luciferase activity ratios.

### 2.13 Scratch wound healing assay

A549 cells (1 × 10^5^) stably expressing SPRTN shRNA, SPRTN overexpression vector, or empty vector were seeded in 6-well plates and treated with BMH-21 (3 µM) or DMSO, or, A549 cells stably expressing SPRTN shRNA or SPRTN overexpression vector were transiently transfected with TAF1C shRNA vector or non-targeting scrambled shRNA control. After 24 h, a uniform scratch was introduced across the confluent monolayer, and wound closure was imaged at 0, 24, 48, and 72 h using an EVOS XL Core microscope (Invitrogen). Wound width was quantified using ImageJ software (NIH, Bethesda, MD, USA) and wound closure was expressed as percentage of initial wound area closed.

### 2.14 Invasion assay

Matrigel (Corning, Corning, NY, USA) diluted 1:5 in serum-free RPMI-1640 medium was applied to transwell inserts (Sigma-Aldrich) and allowed to solidify at 37 °C for 2 h. A549 cells stably expressing empty vector, SPRTN shRNA, or SPRTN overexpression vector, pre-treated with DMSO or BMH-21, or A549 cells stably expressing SPRTN shRNA or SPRTN overexpression vector were transiently transfected with TAF1C shRNA vector or non-targeting scrambled siRNA control were seeded into the upper chamber in serum-free medium, with complete medium (10% FBS) placed in the lower chamber as a chemoattractant. After 24 h, inserts were fixed in cold methanol (15 min), stained with 0.01% crystal violet (20 min, RT), and non-invading cells on the upper surface were removed. Invaded cells were imaged using an EVOS microscope (Invitrogen).

### 2.15 *In vivo* studies

All animal experiments were conducted in accordance with national guidelines and were approved by the Institutional Animal Ethics Committee of the Indian Institute of Science Education and Research, Mohali, Punjab, India (Approval No. IISERM/SAFE/PRT/2024/008; approved 4 May 2024). A549 cells (5 × 10^6^) stably expressing SPRTN shRNA or empty vector control were suspended in PBS and injected subcutaneously into the flanks of 4–6-week-old male CD-1 Foxn1 nude mice (n = 4 per group). Once palpable tumors formed, animals were randomized into treatment groups (Day 1) and received intratumoral Dox (1 mg/kg) or DMSO vehicle every alternate day for 3 weeks. Tumor volume was measured using digital calipers and calculated as (length × width²)/2. Animals were euthanized by cervical dislocation following the treatment period, and tumors were collected for histopathological, immunofluorescence, and molecular analyses.

### 2.16 Histology

Paraffin-embedded xenograft sections (5 µm) were cut using a rotary microtome (Leica, Wetzlar, Germany). For haematoxylin and eosin (H&E) staining, sections were deparaffinized in xylene, rehydrated through a graded ethanol series, stained with haematoxylin (5 min) and eosin (1 min), dehydrated, cleared, and mounted with DPX mountant (HiMedia, Maharashtra, India). Images were captured using an EVOS XL Core microscope (Invitrogen). For immunofluorescence, deparaffinized sections underwent antigen retrieval in 10 mM sodium citrate buffer (pH 6.0), followed by overnight incubation at 4 °C with anti-Ki-67 (1:1000) or anti-Annexin V (1:1000) antibodies. Sections were then incubated with Texas Red- or Alexa Fluor 488-conjugated secondary antibodies (1 h, RT), counterstained with Hoechst 33342, mounted with DPX, and imaged on a Leica DMi8 fluorescence microscope (Leica Microsystems).

### 2.17 Tumor RNA

Total RNA from LUAD tumor and adjacent normal tissue specimens was purchased from the National Cancer Tissue Bank, IIT Madras, Chennai, India.

### 2.18 Statistical analysis

All biological replicates represent independent experiments performed on different cell passages. Technical replicates were averaged prior to statistical analysis, and statistical comparisons were performed using independent biological replicates. All experiments were performed using at least three independent biological replicates, unless otherwise indicated. Data are presented as mean ± SEM. Comparisons between two groups were performed using an unpaired two-tailed Student’s t-test. For experiments involving comparisons among multiple groups, statistical significance was determined using one-way ANOVA followed by Tukey’s multiple-comparisons *post hoc* test. A p-value of less than 0.05 was considered statistically significant. For *in vivo* tumor growth analyses, each animal was treated as an independent biological unit, and growth curves were compared using two-way ANOVA with Geisser-Greenhouse correction.

## 3 Results

### 3.1 SPRTN maintains basal Pol I transcription and promotes cell proliferation

**Figure 1:**
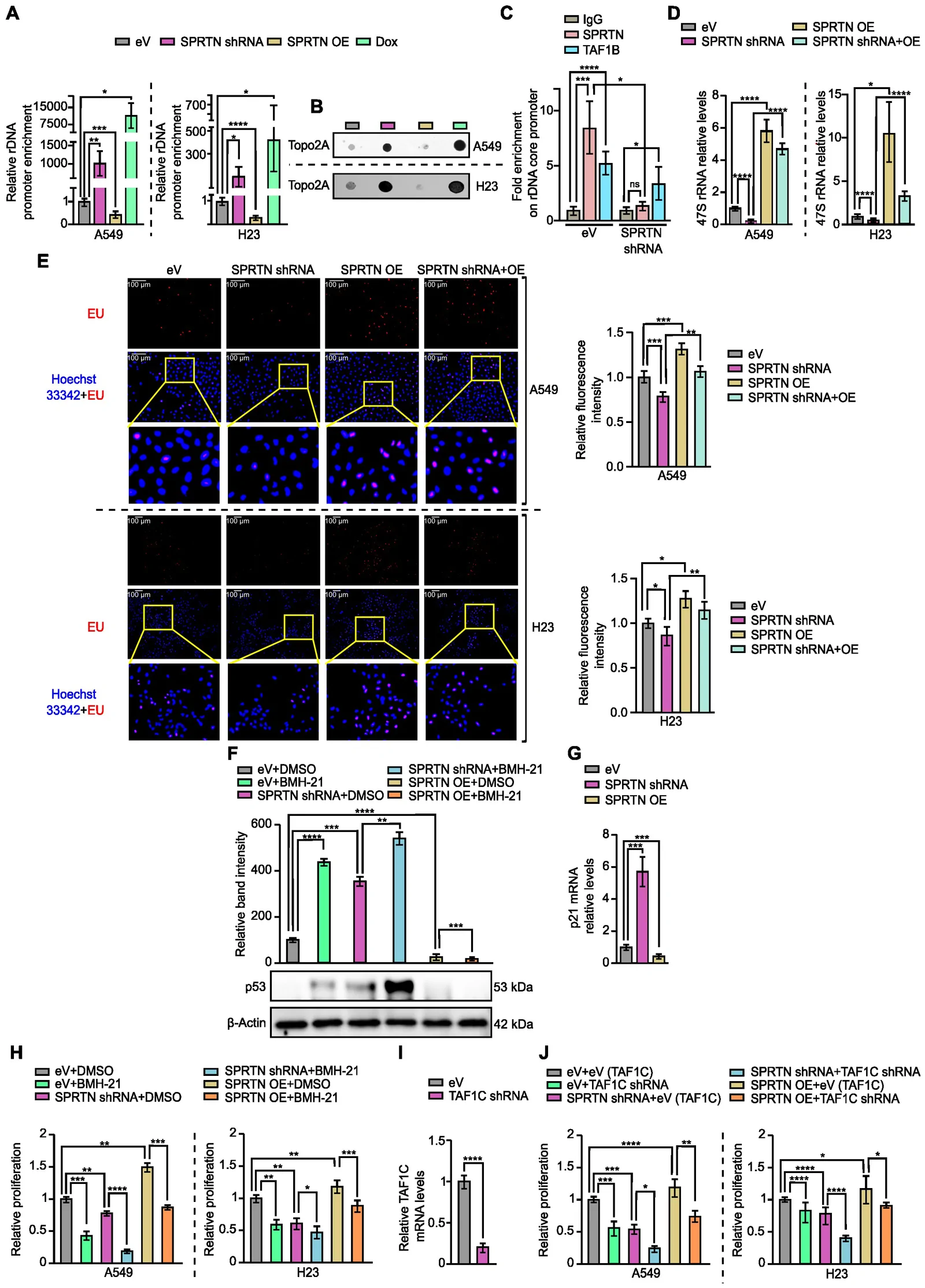
SPRTN limits rDNA-DPC accumulation, regulates rDNA transcription, p53/p21 expression, and cell proliferation. **(A)**. qPCR analysis of the DNA fraction from the RADAR assay showing relative enrichment of the rDNA promoter in SPRTN shRNA, SPRTN-OE, and Dox-treated cells. **(B)**. Representative dot blot of the protein-linked DNA fraction from the RADAR assay demonstrating Topo2A signal intensity in SPRTN shRNA, SPRTN-OE, and Dox-treated cells. **(C)**. ChIP-qPCR showing reduced SPRTN enrichment at the rDNA core promoter in SPRTN knockdown cells compared to control cells, TAF1B signal serves as a positive control for rDNA promoter binding. **(D)**. qPCR quantification of 47S pre-rRNA showing increased levels in SPRTN-OE cells and reduced levels in SPRTN shRNA cells, with restoration after SPRTN reconstitution. **(E)**. Representative fluorescence images showing reduced EU incorporation in SPRTN shRNA cells, enhanced EU incorporation in SPRTN-OE cells, and restoration of the EU signal upon SPRTN reconstitution in shRNA cells, bar graph shows quantification of relative EU fluorescence intensities. **(F)**. Representative immunoblot showing p53 expression in SPRTN shRNA and SPRTN-OE cell lines, alone or in combination with BMH-21, bar graphs indicate band intensities normalized to β-actin signals. **(G)**. qPCR analysis of p21 mRNA levels showing elevation in SPRTN-shRNA cells and reduction upon SPRTN OE. **(H)**. Cell proliferation analysis showing reduced proliferation in SPRTN knockdown cells, further diminished by BMH-21, SPRTN overexpression enhanced proliferation, partially suppressed by BMH-21, bar graph shows quantification of relative proliferation. **(I)**. qPCR analysis confirming TAF1C knockdown in A549 cells following shRNA transfection compared to scrambled control. **(J)**. Cell proliferation analysis showing reduced proliferation upon TAF1C knockdown in SPRTN shRNA and SPRTN-overexpressing cells (n=3 biological replicates). Error bars represent mean ± SEM. *P < 0.05, **P < 0.01, ***P < 0.001, ****P < 0.0001. eV, empty vector; OE, overexpression; shRNA, short hairpin RNA; RADAR, Rapid Approach to DNA Adduct Recovery; ChIP-qPCR, Chromatin Immunoprecipitation-qPCR; rDNA, ribosomal DNA; EU, 5-ethynyl uridine.

Actively transcribed rDNA is vulnerable to transcription-stalling DPCs (24). To assess whether SPRTN contributes to their resolution, we established A549 cells with stable SPRTN knockdown or overexpression, validated by qPCR and immunoblotting (**Supplementary Figures 1A–D**). RADAR assays in SPRTN knockdown and overexpression A549 cells, and in H23 cells with transient SPRTN knockdown or overexpression (validated by qPCR, **Supplementary Figures 1E, F**), revealed that SPRTN depletion increased rDNA promoter recovery within the DPC-bound DNA fraction, whereas SPRTN overexpression reduced this enrichment; Dox treatment served as a DPC-inducing positive control (**Figure 1A**). Consistently, dot-blot analysis of the protein-linked fraction demonstrated parallel changes in Topo2A enrichment (a marker of rDNA-associated DPCs), with elevated Topo2A-DPCs in SPRTN-deficient cells and reduced levels in SPRTN-overexpressing cells, and as expected, Dox markedly increased Topo2A-linked DPC formation (**Figure 1B**). These findings indicate that SPRTN limits promoter-proximal rDNA DPC accumulation under basal conditions.

To assess the functional consequences of SPRTN on Pol I transcriptional output, we performed ChIP-qPCR, which confirmed SPRTN enrichment at the rDNA core promoter above IgG control in A549 empty vector cells, with reduced occupancy upon stable SPRTN knockdown. TAF1B, a core PIC component, served as a positive control and displayed strong rDNA promoter binding (**Figure 1C**). SPRTN overexpression elevated 47S pre-rRNA levels, while SPRTN knockdown reduced 47S pre-rRNA levels, importantly, reconstitution of SPRTN in knockdown cells significantly restored 47S pre-rRNA levels, confirming specificity (**Figure 1D**). EU incorporation assays corroborated these findings at the level of nascent rRNA synthesis (**Figure 1E**; **Supplementary Figure 1G**). To validate the specificity of SPRTN-dependent effects on Pol I transcription, we assessed the effects of acute SPRTN knockdown and overexpression in A549 cells by transient transfection. Transient SPRTN modulation recapitulated the effects on 47S pre-rRNA levels, rRNA synthesis, and proliferation observed in stable cell lines (**Supplementary Figures 1H–L**), suggesting a direct consequence of SPRTN modulation rather than chronic adaptation under SPRTN modulation.

Since Pol I inhibition is known to activate the p53 nucleolar stress pathway (25), we next examined whether SPRTN modulates this nucleolar stress checkpoint. As expected, the Pol I inhibitor BMH-21 (validated by 47S rRNA suppression, **Supplementary Figure 1M**) elevated p53 levels. SPRTN knockdown alone similarly increased p53 expression, and combined SPRTN knockdown and Pol I inhibition produced an additive increase in p53 levels. Conversely, SPRTN overexpression significantly reduced both basal and BMH-21-induced p53 (**Figure 1F**). Consistent with p53 activation, qPCR analysis showed that the p53 target gene p21 was induced upon SPRTN depletion and suppressed by SPRTN overexpression (**Figure 1G**), indicating that SPRTN restrains nucleolar stress signaling by sustaining Pol I transcriptional output. Given that Pol I inhibition disrupts cell proliferation through p53 axis activation (26), we examined whether SPRTN-dependent Pol I transcription regulation influences proliferative capacity. SPRTN knockdown significantly reduced proliferation, an effect further potentiated by the Pol I inhibitor BMH-21, whereas SPRTN overexpression enhanced proliferation and partially rescued BMH-21-induced growth suppression in both A549 and H23 cells (**Figure 1H**). To further confirm Pol I dependence, we performed shRNA-mediated knockdown of TAF1C, a core component essential for Pol I transcription initiation, as an independent genetic approach. TAF1C depletion was confirmed by qPCR (**Figure 1I**) and recapitulated the effects of BMH-21 on SPRTN-dependent proliferation in both SPRTN knockdown and overexpressing cells (**Figure 1J**). Collectively, these findings establish that SPRTN limits basal rDNA-DPC accumulation, sustains Pol I initiation competence at the rDNA core promoter, restrains p53-p21 nucleolar stress signaling, and promotes proliferation in LUAD cells.

### 3.2 SPRTN and PARP-1 maintain rDNA transcription under genotoxic drug stress

**Figure 2:**
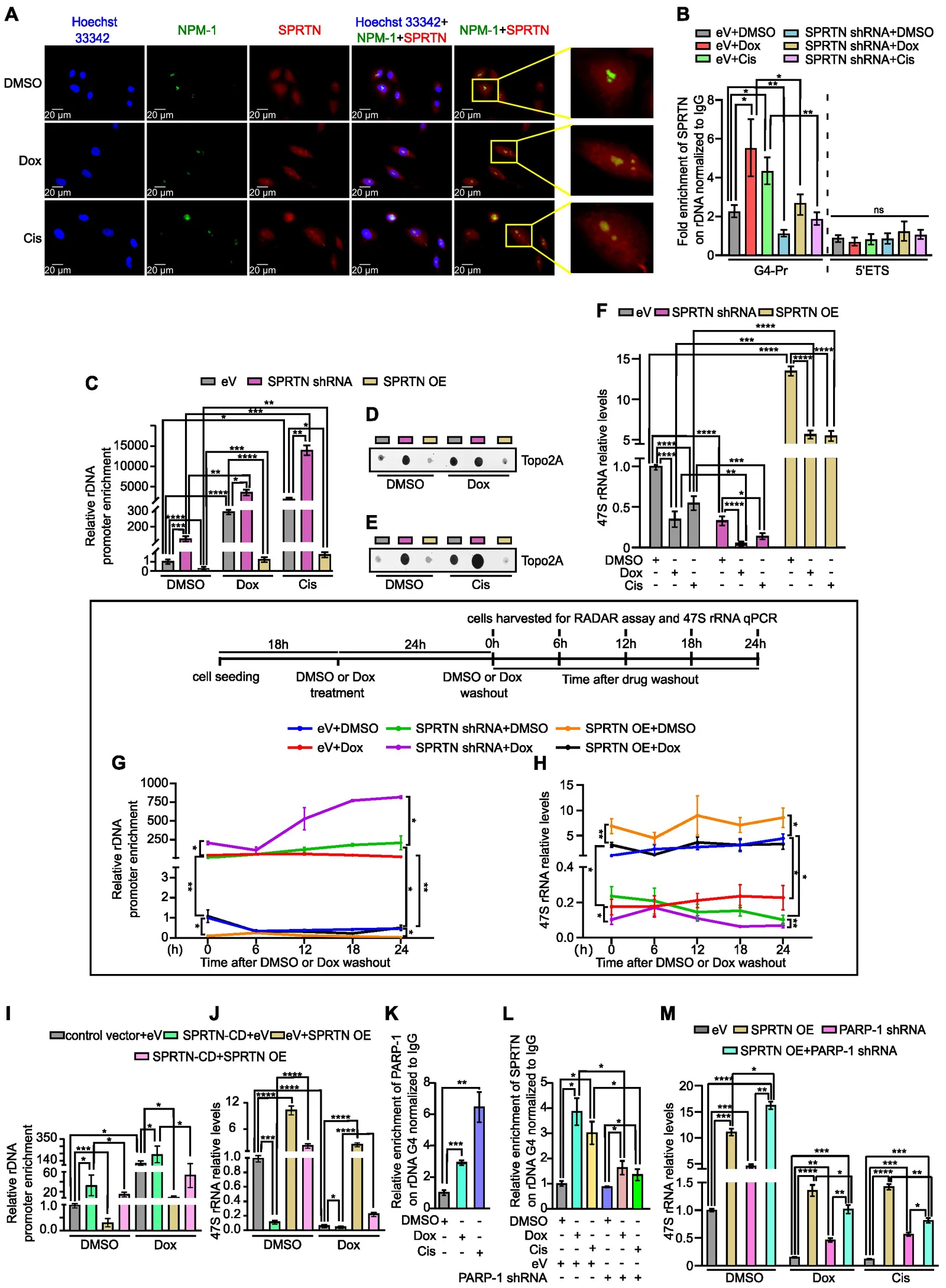
SPRTN and PARP-1 maintain rDNA transcription under genotoxic stress. **(A)** Immunofluorescence images showing SPRTN co-localization with nucleolar NPM-1 and TAF1B following Dox or Cis treatment, compared to DMSO control. **(B)** ChIP–qPCR showing Dox or Cis-induced SPRTN enrichment at the G4-rich rDNA core promoter in A549 empty vector cells, which was reduced upon SPRTN knockdown; no enrichment at the 5′ ETS locus. DMSO, vehicle control. **(C)** qPCR of the DNA fraction from the RADAR assay showing relative enrichment of the rDNA promoter in SPRTN shRNA and SPRTN-OE cells after Dox or Cis, compared to DMSO treatment. **(D)** Representative dot blot of the protein-linked DNA fraction from the RADAR assay showing Topo2A signal intensity in SPRTN shRNA and SPRTN-OE cells following Dox treatment. **(E)** Representative dot blot showing Topo2A signal intensity following Cis treatment. **(F)** qPCR analysis of 47S pre-rRNA showing decreased levels in SPRTN shRNA cells and increased levels in SPRTN-OE cells after Dox or Cis, compared to DMSO. **(G)** RADAR-qPCR showing rDNA-DPC enrichment in A549 empty vector, SPRTN shRNA, and SPRTN-overexpressing cells following doxorubicin washout at timepoints as indicated. DMSO-treated cells serve as control. **(H)** qPCR demonstrating 47S pre-rRNA recovery in the same conditions as panel G at timepoints as indicated. **(I)** RADAR-qPCR analysis showing SPRTN-CD expression increased rDNA-DPC burden under both basal and drug-treated conditions; wild-type SPRTN rescue of SPRTN-CD expressing cells significantly reduced DPC burden. **(J)** qPCR analysis of 47S pre-rRNA levels showing SPRTN-CD reduced Pol I transcriptional output under basal and drug-treated conditions; wild-type SPRTN rescue partially but significantly restored 47S pre-rRNA levels. **(K)** ChIP-qPCR showing PARP-1 recruitment to rDNA G4 promoter regions following Dox, Cis, or DMSO treatment. **(L)** ChIP-qPCR showing SPRTN enrichment at rDNA core promoter G4 regions after Dox or Cis or DMSO treatment, with or without PARP-1 shRNA. **(M)** qPCR analysis of 47S pre-rRNA levels following Dox or Cis treatment in SPRTN-OE cells with or without PARP-1 shRNA. (n=3 biological replicates) Error bars represent mean ± SEM. *P < 0.05, **P < 0.01, ***P < 0.001, ****P < 0.0001, ns, not significant. DPC, DNA-protein crosslink; Dox, doxorubicin; Cis, cisplatin; eV, empty vector; OE, overexpression; G4, G-quadruplex; 5′ ETS, 5′ external transcribed spacer; EU, 5-Ethynyl Uridine; shRNA, short hairpin RNA; Pr, promoter; RADAR, Rapid Approach to DNA Adduct Recovery, SPRTN-CD, SPRTN catalytic inactive mutant.

Genotoxic chemotherapeutics such as Dox and Cis stabilize G4 DNA structures within the rDNA promoter, resulting in transcription-blocking DPCs (27). To determine whether SPRTN associates with and facilitates resolution of these lesions, we first assessed its subcellular localization. Immunofluorescence revealed a marked nucleolar accumulation of SPRTN following Dox or Cis treatment, colocalizing with the nucleolar markers Nucleophosmin-1 (NPM-1) and TAF1B (**Figure 2A**; **Supplementary Figure 2A**). Next, we evaluated SPRTN enrichment at G4-rich regions of the rDNA promoter. ChIP-qPCR in A549 empty vector cells showed a significant increase in SPRTN enrichment at G4-rich rDNA promoter regions in response to Dox or Cis treatment, which was markedly reduced in SPRTN knockdown cells. No enrichment was observed at G4-depleted external transcribed spacer (ETS) loci (**Figure 2B**), indicating selective association of SPRTN with G4-rich rDNA regions rather than the transcribed body of the rDNA unit, consistent with a role in promoter-proximal DPC resolution and Pol I initiation competence.

RADAR analyses further revealed SPRTN-dependent reduction of rDNA-associated DPCs under genotoxic drug stress. qPCR of the DPC-bound DNA fraction showed increased enrichment of the G4-rich rDNA promoter in SPRTN-knockdown cells following Dox or Cis, whereas SPRTN overexpression significantly attenuated this enrichment (**Figure 2C**). Consistently, RADAR dot blots demonstrated elevated Topo2A enrichment in protein-linked DPCs in SPRTN knockdown cells and reduced levels in SPRTN-overexpressing cells in response to Dox or Cis (**Figures 2D, E**). Consistent with altered rDNA-associated DPC burden, Pol I transcriptional output varied proportionally with SPRTN levels. qPCR analysis revealed that Dox and Cis markedly suppressed 47S rRNA levels in control cells, which was further exacerbated by SPRTN knockdown, whereas SPRTN overexpression significantly rescued both basal and drug-induced repression of 47S rRNA levels (**Figure 2F**). Similarly, EU incorporation assays showed that SPRTN depletion exacerbated Dox- or Cis-mediated inhibition of nascent rRNA synthesis, while SPRTN overexpression restored transcriptional activity despite genotoxic stress (**Supplementary Figure 2B**).

To assess kinetics of SPRTN-dependent DPC resolution, we performed RADAR-qPCR at 0, 6, 12, 18, and 24 hours following Dox washout. Under DMSO conditions, SPRTN knockdown elevated rDNA promoter enrichment in the DPC-bound fraction relative to empty vector, while SPRTN overexpression reduced it. Following Dox washout, empty vector cells showed sustained rDNA-DPC enrichment through 12 hours, with a marginal decline thereafter. SPRTN knockdown cells exhibited the highest DPC burden across all timepoints, with a marked increase after 6 hours and sustained elevation through 24 hours. In contrast, SPRTN overexpressing cells displayed markedly lower DPC enrichment at 0 hours and showed progressive reduction throughout the washout period (**Figure 2G**). Consistent with rDNA-DPC dynamics, 47S pre-rRNA levels showed SPRTN-dependent recovery following Dox washout. Under DMSO conditions, SPRTN knockdown cells showed reduced 47S pre-rRNA levels at 0 hours with progressive decline, while SPRTN overexpressing cells maintained markedly elevated 47S pre-rRNA output, consistent with basal Pol I regulation. Following Dox washout, empty vector cells maintained minimal 47S pre-rRNA levels throughout the recovery period, indicating limited transcriptional recovery. SPRTN knockdown cells showed the lowest 47S levels at 0 hours with a transient marginal increase at 6 hours followed by progressive decline, demonstrating that SPRTN depletion further impairs Pol I transcriptional recovery after drug removal. Strikingly, SPRTN-overexpressing cells maintained 47S pre-rRNA levels comparable to the DMSO range throughout the washout period, demonstrating that elevated SPRTN expression sustains Pol I output despite prior genotoxic insult (**Figure 2H**). Collectively, these kinetic data demonstrate that SPRTN determines both the rate of rDNA-DPC resolution and the extent of Pol I transcriptional recovery following genotoxic drug treatment.

To determine whether catalytic activity of SPRTN is required for its effects on rDNA-DPC burden and Pol I transcription, we performed rescue experiments using catalytically inactive SPRTN. RADAR-qPCR showed that SPRTN-CD expression elevated rDNA promoter enrichment in the DPC-bound fraction relative to empty vector under DMSO, while wild-type SPRTN overexpression reduced it; wild-type SPRTN rescue of SPRTN-CD cells significantly reduced rDNA-DPC burden. Under Dox, SPRTN-CD cells accumulated the highest rDNA-DPC levels across all conditions, which were significantly attenuated by wild-type SPRTN rescue (**Figure 2I**). Consistent with these changes, 47S pre-rRNA levels were suppressed in SPRTN-CD cells and elevated in wild-type SPRTN cells under DMSO; wild-type SPRTN rescue significantly restored 47S output. Under Dox, SPRTN-CD cells showed the lowest 47S levels across all conditions, and were significantly restored by wild-type SPRTN rescue (**Figure 2J**). Together, these data establish that SPRTN catalytic activity is essential for promoter-proximal rDNA-DPC resolution and Pol I transcriptional maintenance under basal and genotoxic stress conditions.

Given that PARP-1 transiently inhibits Pol I transcription to facilitate repair of rDNA during genotoxic stress (28), we next assessed whether SPRTN recruitment is influenced by PARP-1. ChIP-qPCR confirmed increased recruitment of PARP-1 to rDNA G4 regions upon Dox or Cis treatment (**Figure 2K**). ChIP-qPCR analysis in PARP-1 knockdown cells showed a significant reduction in SPRTN enrichment on rDNA G4 loci after Dox or Cis exposure (**Figure 2L**), indicating that PARP-1 contributes to Dox- and Cis-induced SPRTN enrichment at G4-rDNA. Accordingly, qPCR analysis revealed that SPRTN overexpression significantly increased basal 47S rRNA levels, while PARP-1 knockdown, consistent with the established role of PARP-1 in DNA damage-induced transient inhibition of Pol I transcription, also elevated basal rRNA synthesis. Combined SPRTN overexpression and PARP-1 depletion produced an additive increase in basal Pol I output. In contrast, upon Dox or Cis treatment, this transcriptional induction was markedly attenuated in PARP-1–knockdown cells and only partially restored by SPRTN overexpression (**Figure 2M**), indicating that PARP-1 contributes to SPRTN-mediated recovery of Pol I transcription under genotoxic stress. These findings demonstrate that SPRTN is enriched at G4-rich rDNA under genotoxic drug stress, in association with PARP-1, and that together they facilitate reduction of basal and drug-induced rDNA DPCs, and sustain Pol I transcription during genotoxic drug challenge.

### 3.3 SPRTN promotes drug tolerance by sustaining Pol I transcription

**Figure 3:**
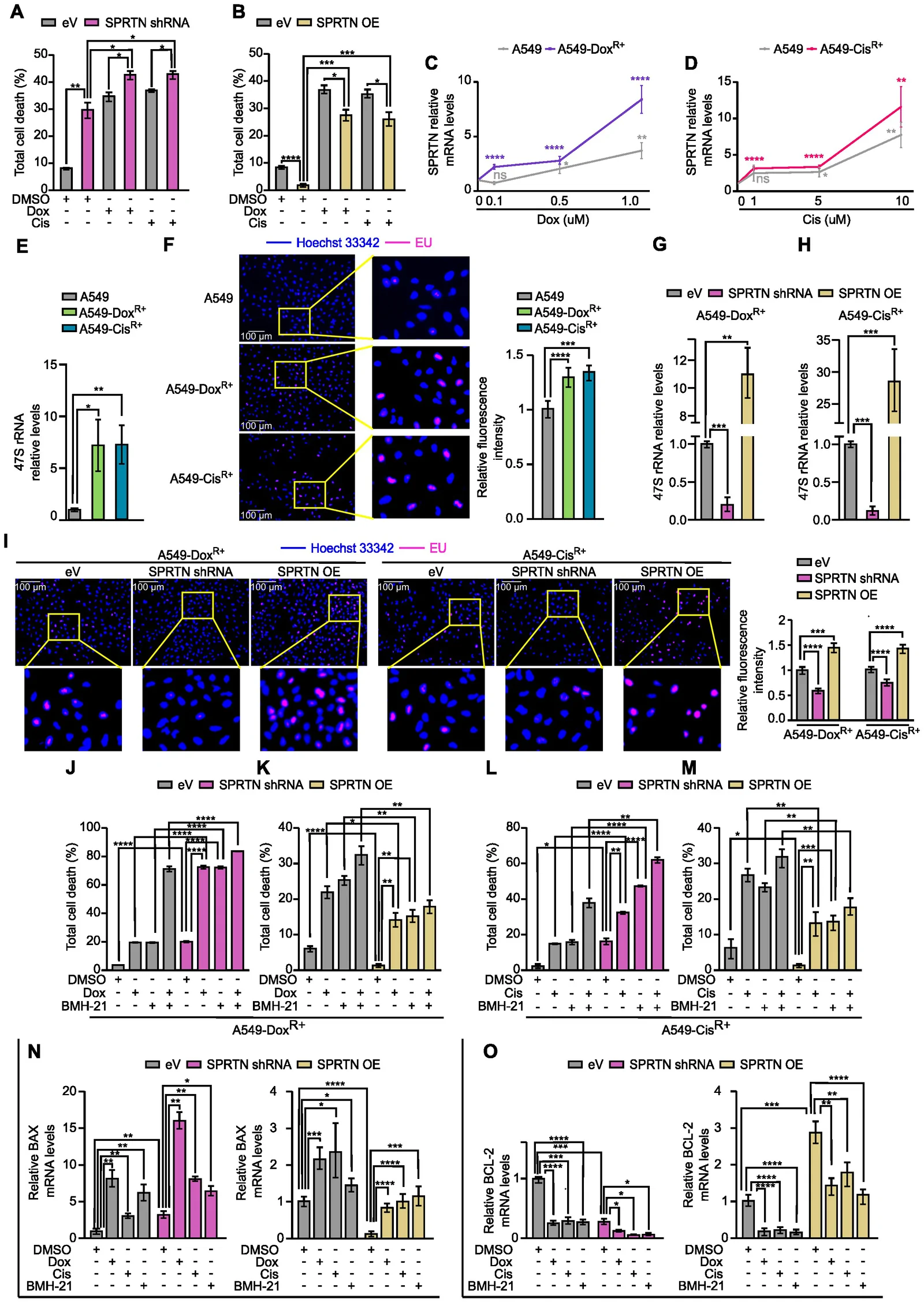
SPRTN modulates apoptosis and drug tolerance in a Pol I transcription-coupled manner. **(A)** Flow-cytometric analysis showing increased basal apoptosis and enhanced apoptotic response to Dox or Cis treatment compared to DMSO in SPRTN-shRNA cells. **(B)** Flow-cytometric analysis showing that SPRTN overexpression reduced basal apoptosis and attenuated Dox or Cis induced apoptosis, compared to DMSO. **(C)** qPCR analysis showing dose-dependent upregulation of SPRTN expression in A549-DoxR^+^ cells treated with increasing Dox concentrations, compared with parental A549 cells. **(D)** qPCR analysis showing dose-dependent upregulation of SPRTN expression in A549-CisR^+^ cells treated with increasing Cis concentrations, compared with parental A549 cells. **(E)** qPCR analysis showing elevated 47S pre-rRNA levels in A549-DoxR^+^ and A549-CisR^+^ cells compared with parental A549 cells. **(F)** EU incorporation assay showing increased nascent RNA synthesis in A549-DoxR^+^ and A549-CisR^+^ cells compared with parental A549 cells; bar graph shows quantification of relative EU fluorescence intensity. **(G)** qPCR analysis showing reduced 47S pre-rRNA levels upon SPRTN knockdown and increased levels upon SPRTN overexpression in A549-DoxR^+^ cells, compared with parental A549 cells. **(H)** qPCR analysis showing reduced 47S pre-rRNA levels upon SPRTN knockdown and increased levels upon SPRTN overexpression in A549-CisR^+^ cells, compared with parental A549 cells. **(I)** Representative EU incorporation images and bar graph quantification showing decreased nascent rRNA synthesis upon SPRTN knockdown and increased nascent rRNA synthesis upon SPRTN overexpression in A549-DoxR^+^ and A549-CisR^+^ cells, compared with empty vector control cells. **(J)**. Flow-cytometric analysis in A549-DoxR^+^ cells showing that SPRTN knockdown increased apoptosis following Dox treatment, with further increase upon BMH-21 co-treatment. **(K)** Flow-cytometric analysis showing that SPRTN overexpression in A549-DoxR^+^ cells dampened apoptosis under Dox or Dox+BMH-21 treatment. **(L)** Flow-cytometric analysis in A549-CisR^+^ cells showing that SPRTN knockdown increased apoptosis after Cis exposure, with an additive increase in apoptosis upon BMH-21 co-treatment. **(M)** Flow-cytometric analysis showing that SPRTN overexpression reduced Cis-induced apoptosis in A549-CisR^+^ cells; combined Cis and BMH-21 treatment produced an additive increase in apoptosis. **(N)** qPCR analysis showing upregulation of the pro-apoptotic gene BAX upon SPRTN knockdown, with reciprocal reduction upon SPRTN overexpression; effects shown with or without Dox, Cis, or BMH-21 treatment relative to DMSO. **(O)** qPCR analysis showing downregulation of the anti-apoptotic gene BCL-2 upon SPRTN knockdown, with reciprocal increase upon SPRTN overexpression; effects shown with or without Dox, Cis, or BMH-21 treatment relative to DMSO (n=3 biological replicates). Error bars represent mean ± SEM. *P < 0.05, **P < 0.01, ***P < 0.001, ****P < 0.0001. EU, 5-Ethynyl Uridine; OE, overexpression; shRNA, short hairpin RNA; A549-DoxR^+^/A549-CisR^+^, doxorubicin- or cisplatin-resistant A549 lines; Dox, doxorubicin; Cis, cisplatin; eV, empty vector.

Given the role of SPRTN in maintaining rDNA transcription under genotoxic drug stress, we first assessed its influence on drug response. A549 cells with stable SPRTN knockdown or overexpression were treated with Dox or Cis. SPRTN knockdown significantly increased basal apoptosis and further augmented drug-induced cell death, whereas SPRTN overexpression markedly reduced both basal and drug-induced apoptosis (**Figures 3A, B**; **Supplementary Figures 3A, B**). Similar effects were observed in H23 cells (**Supplementary Figures 3C, D**). Next, to evaluate whether SPRTN-driven Pol I transcription contributes to survival in drug-resistant cells, we employed Dox-resistant A549-DoxR^+^ and Cis-resistant A549-CisR^+^ cells, which displayed a significant reduction in apoptosis in response to Dox or Cis, respectively, and a marked induction of canonical drug-resistance genes (ABCG2, MRP2, ABCB1, ZEB1) relative to parental controls (**Supplementary Figures 4A–F**). SPRTN expression was significantly upregulated in A549-DoxR^+^ (**Figure 3C**) and A549-CisR^+^ (**Figure 3D**) cells, in a dose-dependent manner. Consistent with this SPRTN upregulation, 47S rRNA levels were elevated in A549-DoxR^+^ and A549-CisR^+^ cells (**Figures 3E, F**; **Supplementary Figure 5A**). Moreover, SPRTN knockdown abrogated this elevated rRNA output, whereas SPRTN overexpression further enhanced rRNA synthesis (**Figures 3G–I**; **Supplementary Figure 5B**). SPRTN knockdown increased basal and drug-induced apoptosis in both A549-DoxR^+^ and A549-CisR^+^ cells, and co-treatment with the Pol I inhibitor BMH-21 further increased apoptosis. In contrast, SPRTN overexpression significantly reduced apoptosis despite BMH-21 treatment (**Figures 3J–M**; **Supplementary Figures 6A, B**).

As SPRTN sustains Pol I transcription and restrains p53 activation, we next examined whether SPRTN influences apoptosis by regulating the downstream BAX/BCL-2 axis of the nucleolar stress response. SPRTN knockdown upregulated the pro-apoptotic factor BAX under basal conditions, an effect further enhanced by Dox or Cis treatment, whereas SPRTN overexpression reduced both basal and drug-induced BAX levels. Consistent with Pol I-linked apoptotic regulation, BMH-21 elevated BAX expression, an effect augmented by SPRTN knockdown and dampened by SPRTN overexpression (**Figure 3N**). The anti-apoptotic factor BCL-2 showed opposite changes. SPRTN knockdown decreased BCL-2 levels, with further suppression upon Dox or Cis treatment, while SPRTN overexpression increased BCL-2 in both basal and drug-treated conditions. BMH-21 reduced BCL-2 expression, with stronger suppression in SPRTN-knockdown cells and attenuation in SPRTN-overexpressing cells (**Figure 3O**). Together, these results indicate that SPRTN supports Pol I-linked survival under genotoxic drug stress by maintaining rDNA transcription and modulating BAX/BCL-2-dependent apoptosis.

### 3.4 SPRTN-Pol I axis promotes migration and invasion

**Figure 4:**
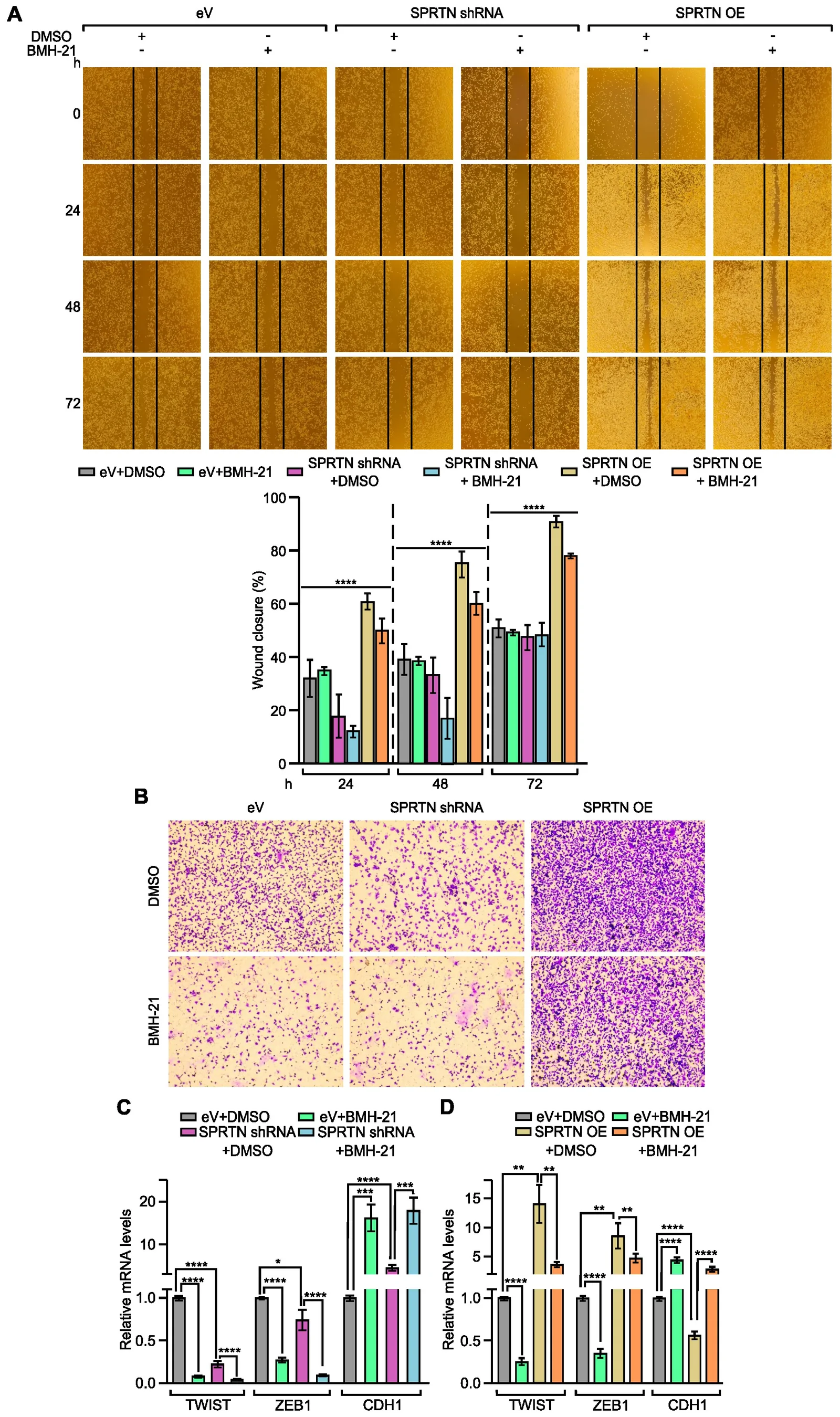
SPRTN promotes migration and invasion in a Pol I-linked manner. **(A)** Wound-healing assay showing reduced migration in SPRTN shRNA cells, further diminished upon BMH-21 treatment, and increased migration in SPRTN-OE cells, with partial attenuation by BMH-21, bar graphs show quantification of wound closure area. **(B)** Transwell invasion assay showing decreased invasion upon SPRTN knockdown, further diminished upon BMH-21 treatment, and increased invasion upon SPRTN OE, partially attenuated by BMH-21. **(C)** qPCR analysis of EMT marker expression in SPRTN knockdown cells, with or without BMH-21 treatment. **(D)** qPCR analysis of EMT marker expression in SPRTN-overexpressing cells, with or without BMH-21 treatment (n=3 biological replicates). Error bars represent mean ± SEM. *P < 0.05, **P < 0.01, ***P < 0.001, ****P < 0.0001. eV, empty vector; OE, overexpression; EMT, epithelial-mesenchymal transition; shRNA, short hairpin RNA.

Pol I transcription has been linked to EMT in LUAD (7, 8, 29). To determine whether the SPRTN-Pol I axis regulates these phenotypes, we assessed migration, invasion, and EMT marker expression in SPRTN-knockdown and SPRTN-overexpressing cells, with or without BMH-21. In wound closure assays, SPRTN depletion markedly delayed wound closure under both basal and Pol I-inhibited conditions, whereas SPRTN overexpression accelerated closure, an effect partially suppressed by BMH-21 (**Figure 4A**). Matrigel invasion assays showed a similar pattern. SPRTN knockdown significantly reduced invasion, further diminished by BMH-21, while SPRTN overexpression increased invasion, an effect attenuated upon Pol I inhibition (**Figure 4B**). We next examined EMT marker expression. SPRTN knockdown decreased the mesenchymal regulators TWIST and ZEB1 while upregulating the epithelial marker CDH1; BMH-21 treatment further enhanced this epithelial shift. Conversely, SPRTN overexpression elevated TWIST and ZEB1 while suppressing CDH1, and these mesenchymal shifts were attenuated by BMH-21 (**Figures 4C, D**). To corroborate the Pol I specificity of SPRTN-driven migratory and invasive phenotypes through a genetic approach, TAF1C shRNA-mediated depletion in SPRTN knockdown and overexpressing A549 cells produced concordant effects on wound closure, invasion, and EMT marker expression as observed with BMH-21 (**Supplementary Figures 7A–D**). Together, these data establish that the SPRTN-Pol I axis promotes migration, invasion, and mesenchymal gene expression in LUAD cells.

### 3.5 SPRTN is upregulated in lung adenocarcinoma and regulated by ZEB1

**Figure 5:**
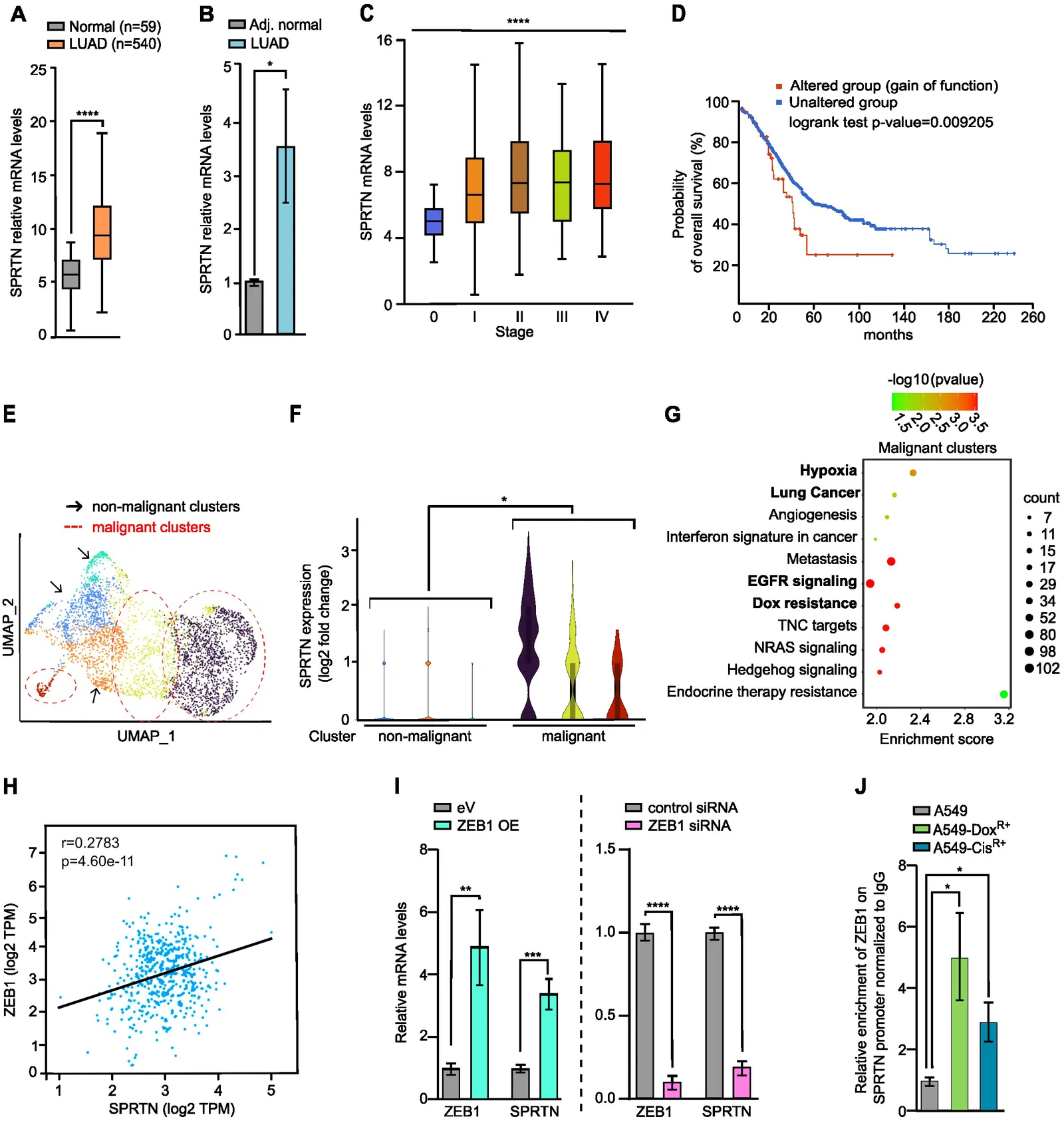
SPRTN is upregulated in LUAD and transcriptionally regulated by ZEB1. **(A)** TCGA analysis showing upregulation of SPRTN in LUAD tumors compared with adjacent normal lung tissue. **(B)** qPCR analysis showing increased SPRTN expression in LUAD tumor samples relative to adjacent normal tissue. **(C)** Stagewise analysis showing progressive increase in SPRTN expression with advancing LUAD pathological stage. **(D)** Kaplan-Meier survival analysis showing that high SPRTN expression is associated with reduced overall survival in LUAD. **(E)** UMAP visualization of LUAD scRNA-seq data showing distinct clusters of malignant and non-malignant cells. **(F)** Violin plots showing differential expression of SPRTN across malignant and non-malignant clusters, with significantly higher levels in malignant clusters. **(G)** GO enrichment analysis of SPRTN-high clusters revealing pathways associated with chemoresistance, EMT, and therapeutic resistance. **(H)** Scatter plot showing a positive correlation between ZEB1 and SPRTN expression in LUAD datasets. **(I)** qPCR analysis showing that ZEB1 overexpression increased SPRTN expression in LUAD cells, and reduced SPRTN expression upon ZEB1 siRNA transfection compared to control. **(J)** ChIP-qPCR showing ZEB1 enrichment at the SPRTN promoter in A549-DoxR^+^ and A549-CisR^+^ cells (n=3 biological replicates). Error bars represent mean ± SEM. *P < 0.05, **P < 0.01, ***P < 0.001, ****P < 0.0001. LUAD, lung adenocarcinoma; qPCR, quantitative PCR; scRNA-seq, single-cell RNA sequencing; UMAP, Uniform Manifold Approximation and Projection; GO, Gene Ontology; DoxR^+^/CisR^+^, drug-resistant A549 derivatives.

Given that Pol I transcription is hyperactivated in LUAD, which can impose a substantial DPC burden on rDNA, we examined SPRTN expression in LUAD. Analysis of TCGA datasets revealed a marked upregulation of SPRTN in LUAD tumors compared with adjacent normal tissues (**Figure 5A**), which was independently confirmed by qPCR in LUAD tumor samples (**Figure 5B**). Stratification by clinical stage revealed a progressive increase in SPRTN expression with advancing disease (**Figure 5C**). Kaplan-Meier analysis demonstrated that high SPRTN expression correlated with reduced overall survival (**Figure 5D**), indicating its prognostic relevance in LUAD. Analysis of LUAD scRNA-seq datasets showed substantial intratumoral heterogeneity in SPRTN expression. UMAP clustering identified multiple malignant and non-malignant clusters (**Figure 5E**). Quantitative comparison of these clusters demonstrated significantly higher SPRTN transcript levels in malignant clusters (**Figure 5F**). Moreover, GO analysis revealed that SPRTN-high clusters were enriched for hallmark pathways associated with chemoresistance, EMT, and therapeutic resistance (**Figure 5G**), indicating selective activation of SPRTN in aggressive LUAD cell states.

To elucidate the mechanisms underlying SPRTN upregulation, we scanned transcription factor binding motifs in the SPRTN promoter and found multiple ZEB1-binding motifs within its proximal promoter (**Supplementary Figure 8**). Across LUAD transcriptomic datasets, ZEB1 and SPRTN showed a modest but statistically significant positive correlation (**Figure 5H**). Consistent with this, ZEB1 overexpression significantly increased SPRTN expression, conversely, siRNA-mediated ZEB1 knockdown significantly reduced SPRTN mRNA levels compared to control (**Figure 5I**). Given that ZEB1 is a master EMT transcription factor that drives drug resistance and is elevated in A549-DoxR^+^ and A549-CisR^+^ cells, we examined whether ZEB1 directly regulates SPRTN. ChIP-qPCR confirmed ZEB1 binding at the SPRTN promoter in A549-DoxR^+^ and A549-CisR^+^ cells (**Figure 5J**). These findings demonstrate that ZEB1 transcriptionally activates SPRTN, and that this regulatory axis is reinforced in drug-tolerant LUAD.

### 3.6 miR-195-5p post-transcriptionally represses SPRTN and modulates drug response

**Figure 6:**
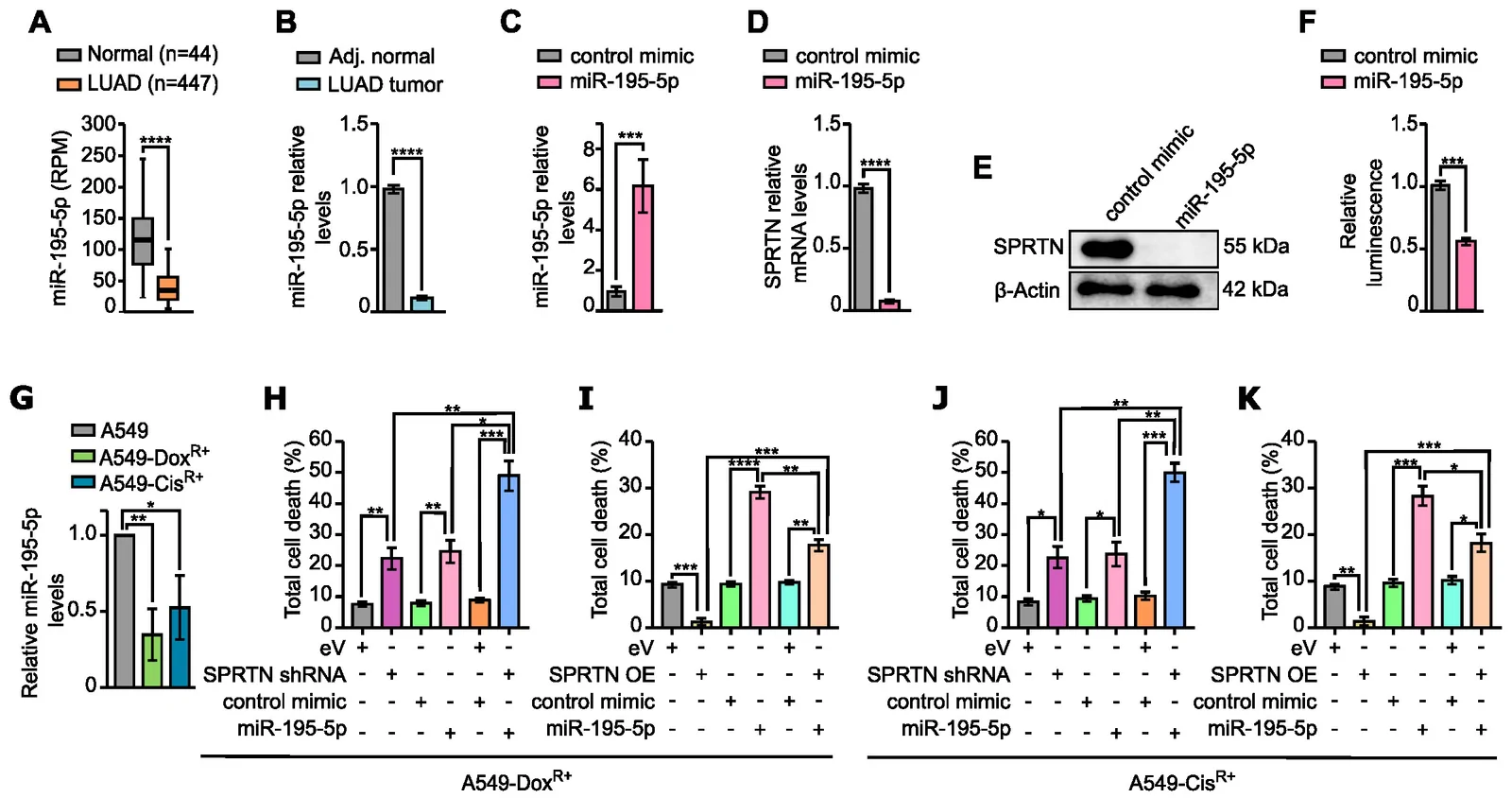
miR-195-5p post-transcriptionally represses SPRTN and modulates drug response in LUAD. **(A)** TCGA analysis showing downregulation of miR-195-5p expression in LUAD tumors compared with adjacent normal lung tissue. **(B)** qPCR analysis showing miR-195-5p downregulation in LUAD tumor samples. **(C)** qPCR analysis confirming increased miR-195-5p expression after transfection with a miR-195-5p mimic. **(D)** qPCR analysis showing that miR-195-5p mimic reduced SPRTN mRNA levels. **(E)** Representative immunoblot showing reduced SPRTN protein levels upon ectopic expression of miR-195-5p mimic. **(F)** Luciferase reporter assay showing reduced activity of SPRTN 3′UTR reporter upon miR-195-5p mimic transfection, confirming direct post-transcriptional repression. **(G)** qPCR analysis showing reduced miR-195-5p expression in A549-DoxR^+^ and A549-CisR^+^ cells relative to parental A549 cells. **(H)** Flow-cytometric analysis showing that SPRTN knockdown and miR-195-5p mimic each increased apoptosis, with combined treatment producing an additive apoptotic response, in A549-DoxR^+^ cells. **(I)** Flow-cytometric analysis showing that SPRTN overexpression attenuated miR-195-5p-induced apoptosis in A549-DoxR^+^ cells. **(J)** Flow-cytometric analysis in A549-CisR^+^ cells showing additive apoptosis upon combined SPRTN knockdown and miR-195-5p mimic transfection. **(K)** Flow-cytometric analysis showing attenuation of miR-195-5p-induced apoptosis upon SPRTN overexpression in A549-CisR^+^ cells (n=3 biological replicates). Data represent mean ± SEM. *P < 0.05, **P < 0.01, ***P < 0.001, ****P < 0.0001. LUAD, lung adenocarcinoma; OE, overexpression; 3′UTR, 3′ untranslated region; DoxR^+^/CisR^+^, doxorubicin- or cisplatin-resistant A549 cells; Dox, doxorubicin; Cis, cisplatin; eV, empty vector.

miRNA-mediated regulatory circuits are key determinants of LUAD chemoresistance (30). To determine whether SPRTN is post-transcriptionally regulated, we screened chemoresistance-associated miRNAs and identified miR-195-5p, previously characterized for its role in drug responsiveness in LUAD (31), as a high-confidence candidate predicted to target the SPRTN 3′UTR (**Supplementary Figures 9A, B**). TCGA analysis and qPCR of LUAD tumor samples demonstrated that miR-195-5p is significantly downregulated in LUAD (**Figures 6A, B**), with TCGA analysis also revealing an inverse correlation between miR-195-5p and SPRTN expression (**Supplementary Figure 9C**). Ectopic expression of miR-195-5p mimics, confirmed by qPCR (**Figure 6C**), reduced SPRTN mRNA and protein levels (**Figures 6D, E**) and significantly suppressed SPRTN 3′UTR luciferase reporter activity, indicating direct post-transcriptional repression (**Figure 6F**). miR-195-5p expression was significantly downregulated in A549-DoxR^+^ and A549-CisR^+^ cells, corresponding to elevated SPRTN expression (**Figure 6G**). In A549-DoxR^+^ cells, ectopic expression of miR-195-5p mimics markedly increased apoptosis, exceeding the effect of SPRTN knockdown alone, while combined miR-195-5p mimic and SPRTN knockdown produced an additive apoptotic response (**Figure 6H** and **Supplementary Figure 9D**). Conversely, SPRTN overexpression suppressed basal apoptosis and significantly attenuated miR-195-5p–induced apoptosis (**Figure 6I**; **Supplementary Figure 9D**). Similarly, in A549-CisR^+^ cells, miR-195-5p mimic transfection significantly enhanced apoptosis beyond that induced by SPRTN knockdown, and dual suppression further amplified this effect (**Figure 6J** and **Supplementary Figure 9E**). SPRTN overexpression mitigated miR-195-5p-mediated apoptosis (**Figure 6K**; **Supplementary Figure 9E**), indicating functional antagonism between SPRTN and miR-195-5p. Collectively, these data establish miR-195-5p as a post-transcriptional repressor of SPRTN in LUAD, and suggest that downregulation of miR-195-5p in resistant LUAD relieves post-transcriptional suppression of SPRTN and promotes drug tolerance.

### 3.7 SPRTN knockdown suppresses tumor growth and produces additive antitumor effects with doxorubicin *in vivo*

**Figure 7:**
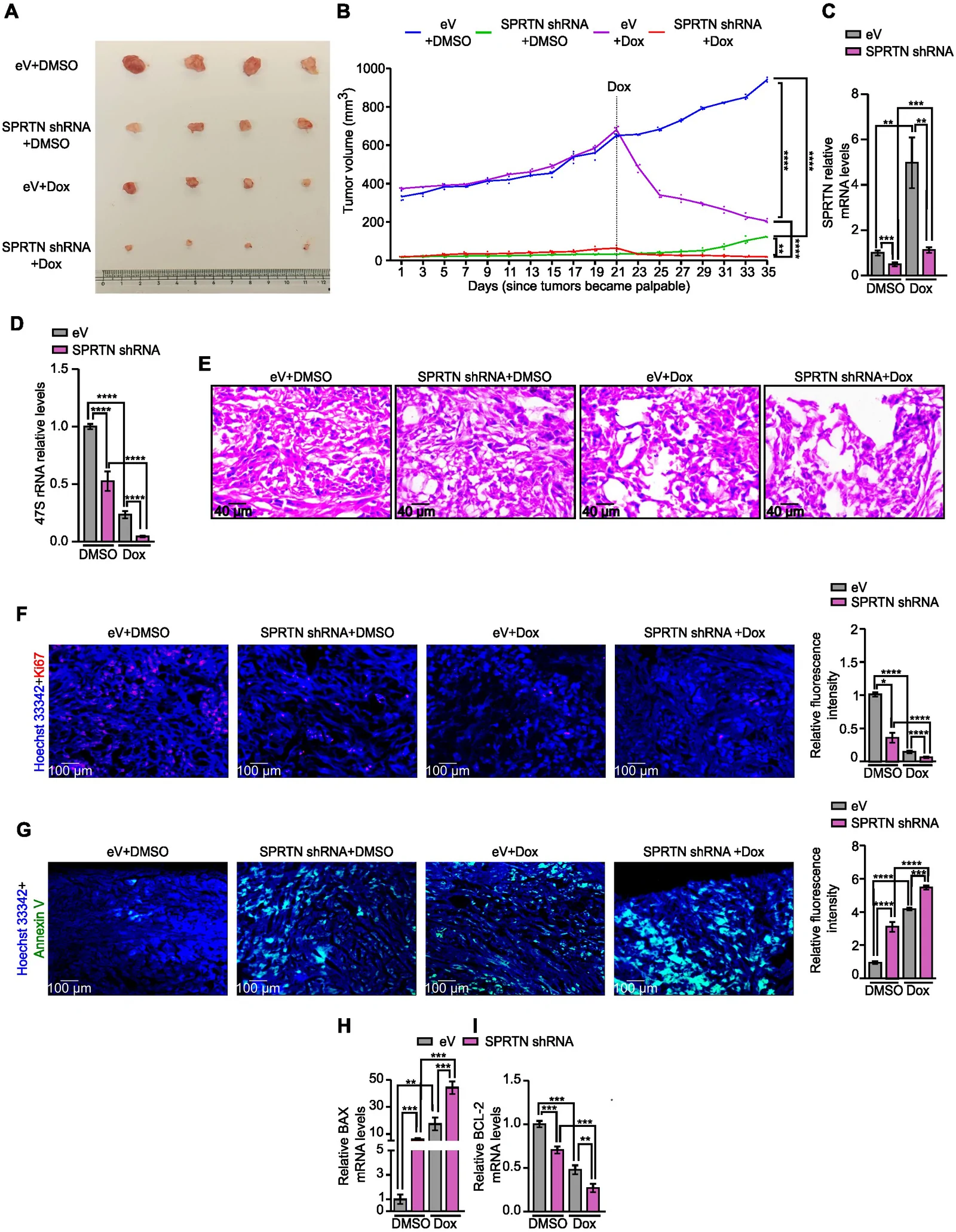
SPRTN knockdown suppresses tumor growth and produces additive antitumor effects with doxorubicin *in vivo*. **(A)** Xenograft images showing reduced tumor size in SPRTN shRNA and Dox-treated groups compared to empty vector control, with further reduction in the combined SPRTN shRNA and Dox group. **(B)** Tumor volume measurements across eV+DMSO, SPRTN shRNA+DMSO, eV + Dox, and SPRTN shRNA + Dox treatment groups. **(C)** qPCR analysis confirming sustained SPRTN depletion in xenograft tissues. **(D)** qPCR analysis showing decreased 47S pre-rRNA across groups, with marked suppression in the combined SPRTN shRNA and Dox tumors. **(E)** H&E staining showing reduced cellularity and disrupted tumor architecture in SPRTN shRNA-expressing and Dox treated tumors, most pronounced in the combination group. **(F)** Ki67 immunofluorescence showing decreased proliferation in SPRTN shRNA and Dox-treated groups, with the lowest signal in the combination group; bar graphs show relative Ki67 fluorescence intensity. **(G)** Annexin-V staining showing increased apoptosis upon SPRTN shRNA and Dox, with further apoptosis in the combined SPRTN shRNA and Dox group; bar graphs show relative Annexin-V fluorescence. **(H)** qPCR analysis showing upregulation of pro-apoptotic BAX in SPRTN shRNA tumors, with further elevation in the combined SPRTN shRNA and Dox tumors. **(I)** qPCR analysis showing downregulation of anti-apoptotic BCL-2, with the marked decrease in the combination group (n=4 mice). Error bars represent mean ± SEM. *P < 0.05, **P < 0.01, ***P < 0.001, ****P < 0.0001. eV, empty vector; shRNA, short hairpin RNA; H&E, hematoxylin-eosin; Dox, doxorubicin.

Given that SPRTN sustains Pol I transcription and promotes proliferation and drug tolerance *in vitro*, we next evaluated whether SPRTN depletion influences tumor growth and Dox response *in vivo*. A549 xenografts stably expressing SPRTN shRNA or control vector were established subcutaneously and treated with Dox or DMSO vehicle once tumors became palpable. SPRTN shRNA xenografts exhibited slower tumor establishment and smaller baseline volumes at treatment initiation compared to empty vector controls, consistent with the antiproliferative effects of SPRTN depletion observed *in vitro*. SPRTN knockdown alone significantly suppressed tumor growth relative to empty vector controls, and Dox treatment produced further reduction in tumor volume in both groups. Repeated-measures two-way ANOVA with Geisser–Greenhouse correction revealed significant effects of treatment (F (3, 12) = 23796, P < 0.0001), time (F = 350.6, P < 0.0001), and their interaction (F = 547.7, P < 0.0001), indicating distinct tumor growth dynamics across conditions (**Figures 7A, B**).

qPCR analysis confirmed sustained SPRTN depletion in xenograft tissues (**Figure 7C**), accompanied by reduced 47S rRNA levels, indicating SPRTN-dependent suppression of Pol I transcription *in vivo* (**Figure 7D**). Histological analysis revealed disrupted tissue architecture and reduced cellular density in SPRTN-deficient tumors, while combined SPRTN knockdown and Dox treatment produced extensive necrosis, stromal expansion, and cellular degradation (**Figure 7E**). Ki67 immunostaining showed reduced proliferation in SPRTN-knockdown tumors, further diminished upon Dox co-treatment (**Figure 7F**; **Supplementary Figure 10A**). Annexin V staining demonstrated increased apoptosis upon SPRTN knockdown, further enhanced by Dox treatment (**Figure 7G**, **Supplementary Figure 10B**). Pro-apoptotic BAX was elevated and anti-apoptotic BCL-2 was reduced in SPRTN-knockdown and Dox-treated tumors, with cumulative changes observed in the combined group (**Figures 7H, I**). Collectively, these findings demonstrate that SPRTN knockdown suppresses Pol I transcription and reduces tumor burden *in vivo*, and that Dox treatment produces further antitumor effects in SPRTN-depleted tumors, supporting SPRTN as a candidate therapeutic target in LUAD.

## 4 Discussion

This study uncovers a previously unrecognized function of the DPC repair factor SPRTN, acting through its protease activity, as a key effector of rDNA DPC repair and Pol I transcriptional homeostasis in LUAD. SPRTN preserves basal rRNA synthesis and facilitates transcriptional recovery after genotoxic stress by Dox and Cis, thereby maintaining the ribosomal output essential for malignant proliferation and cell survival. Pol I inhibition abrogates the proliferative, EMT-associated effects and drug tolerance associated with SPRTN, establishing that these oncogenic phenotypes depend, at least in part, on sustained Pol I activity. Together, our findings define a SPRTN-Pol I axis in which SPRTN acts as a nucleolar stress-responsive factor that preserves rDNA transcription and supports tumor growth and drug tolerance.

Recent studies established that transcription-coupled DPC repair in Pol II-transcribed genes functions through a CSA/CSB–CRL4CSA–proteasome pathway and does not require SPRTN (32, 33). Our work identifies a distinct DPC response at Pol I-transcribed rDNA. rDNA promoters differ fundamentally from Pol II genomic loci; they operate within the nucleolus, sustain exceptionally high transcriptional flux, display repetitive architecture enriched in G-quadruplex motifs, and experience substantial torsional stress, conditions that favor promoter-proximal DPC accumulation. Doxorubicin and cisplatin, which preferentially stabilize G-quadruplex structures, selectively damage the G4-enriched rDNA promoter under conditions of high transcriptional flux. SPRTN-mediated DPC resolution at this site restores Pol I initiation competence, the rate-limiting determinant of rRNA output, thereby maintaining transcriptional output without requiring activity across the full transcribed unit. Under genotoxic challenge, SPRTN is robustly enriched at G4-rich rDNA promoters in association with PARP-1, where it facilitates DPC resolution and preserves Pol I transcriptional output. These findings support a model in which DPC repair is polymerase- and chromatin-context dependent. The rDNA-SPRTN–PARP-1 axis operates specifically at the G4-enriched promoter to maintain initiation competence, distinct from canonical Pol II–coupled pathways.

The role of SPRTN in replication-coupled DPC repair is well established (34, 35). Our findings extend its function to transcriptional maintenance at the rDNA locus. Modulating SPRTN levels proportionally alters rRNA abundance and proliferation, effects attenuated by the Pol I inhibitor BMH-21, confirming a functional SPRTN-Pol I axis. Inhibition of Pol I transcription is known to trigger nucleolar stress, activating the p53-p21 cascade, and inducing cell-cycle arrest (36). Within this circuitry, SPRTN acts as a buffer, its depletion activates p53-p21 expression, while overexpression attenuates p53 induction under Pol I inhibition. Thus, SPRTN safeguards rDNA transcriptional competence against DPC-induced suppression, maintaining proliferative continuity by restraining the nucleolar stress-dependent p53-p21 checkpoint.

Dox and Cis preferentially target rDNA, stabilizing G4 structures, inducing DPCs, and triggering nucleolar stress, mechanisms that underlie a significant component of their cytotoxic action (14–18). Under genotoxic stress, PARP-1 accumulates at rDNA G4 hotspots and exerts a dual function, it imposes a transient transcriptional brake on Pol I, a protective response that limits collisions with stalled complexes, and promotes DPC resolution through PARylation, which remodels local chromatin and recruits repair factors (28, 37). Consistent with this, SPRTN is rapidly enriched to G4-rich rDNA promoters in response to Dox and Cis, where it contributes to DPC reduction and transcriptional recovery. These findings support a model in which PARP-1 accumulation at damaged rDNA facilitates SPRTN enrichment and DPC resolution, thereby enabling Pol I transcriptional recovery after genotoxic insult. However, the mechanistic basis for PARP-1 knockdown-induced elevation of Pol I output, whether reflecting loss of catalytic PARylation activity, scaffolding function at rDNA, or compensatory transcriptional adaptation, warrants further investigation using PARP catalytic inhibitors and catalytically inactive PARP-1 rescue approaches. SPRTN upregulation in A549-DoxR^+^ and A549-CisR^+^ cells was associated with elevated rRNA synthesis, sustaining Pol I output despite persistent genotoxic stress. Collectively, these results establish SPRTN as a key factor linking nucleolar damage repair, Pol I transcriptional resilience, and drug tolerance in LUAD.

EMT, driven by transcription factors such as ZEB1, underlies metastasis and therapy resistance by rewiring epigenetic, transcriptional, translational, and cell-cycle programs in cancer (38). EMT initiation coincides with Pol I transcriptional activation, whereas inhibition of rRNA synthesis disrupts EMT and suppresses metastasis (29). Our previous work demonstrated that miRNA-mediated inhibition of Pol I transcription reduces LUAD cell migration and downregulates ZEB1 (7), while LINC01116-driven invasion and EMT marker expression require Pol I activation, establishing Pol I-linked control of EMT regulators (8). In this study, we identified SPRTN as a direct transcriptional target of ZEB1, functioning as a nucleolar effector that preserves rDNA integrity and sustains Pol I activity under genotoxic stress. By integrating the rDNA damage response with Pol I transcriptional resilience, SPRTN supports rRNA-dependent ribosome biogenesis and EMT persistence. Collectively, the ZEB1-SPRTN-Pol I axis defines a regulatory circuit linking nucleolar repair, proliferation, EMT and drug tolerance in LUAD. SPRTN expression is post-transcriptionally repressed by miR-195-5p, a miRNA previously implicated in EMT suppression and reversal of drug resistance (31, 39, 40). Consistent with its tumor suppressive role, miR-195-5p levels were markedly reduced in A549-DoxR^+^ and A549-CisR^+^ cells, inversely correlating with SPRTN upregulation. Restoration of miR-195-5p, alone or in combination with SPRTN silencing, markedly enhanced apoptosis in resistant cells, indicating functional antagonism between the two. Collectively, this dual-layered regulation positions SPRTN as a convergence point linking EMT signaling, Pol I transcriptional output, and adaptive drug response in LUAD.

## 5 Conclusions

Overall, this work reveals that SPRTN is a nucleolar stress-responsive DPC repair factor and transcriptional effector of ZEB1 that integrates genotoxic drug-induced rDNA damage repair, Pol I transcriptional resilience, and EMT-driven survival in LUAD. By coupling ZEB1-mediated transcriptional activation with miR-195-5p-dependent post-transcriptional control, SPRTN orchestrates a regulatory axis that sustains rDNA integrity, dampens p53-p21 signaling, and reinforces adaptation to genotoxic drug stress. These findings establish the SPRTN-Pol I circuit as a key determinant of nucleolar homeostasis and drug tolerance. However, future studies in non-replicating or replication-arrested cell systems will be important to distinguish direct rDNA-transcriptional effects of SPRTN from indirect consequences of genome-wide DPC accumulation and replication stress. Further, future validation in immunocompetent and patient-derived models will be important to further evaluate the therapeutic relevance of SPRTN targeting. SPRTN remains an understudied therapeutic target, with no selective inhibitors currently available. Our findings position SPRTN as a targetable vulnerability in drug-tolerant tumors, providing a mechanistic basis for the development of SPRTN-targeting strategies. Future development of selective SPRTN inhibitors and their evaluation in combination with standard-of-care genotoxic agents will be an important next step in translating these findings.

## Data Availability

The original contributions presented in the study are included in the article/**Supplementary Material**. Further inquiries can be directed to the corresponding authors.
