# Supplementary material for: SPRTN preserves RNA polymerase I transcription under genotoxic drug stress in lung adenocarcinoma
Source: Front Oncol. 2026 Aug 17;16:1917106. doi: 10.3389/fonc.2026.1917106 (PMC13526629; doi:10.3389/fonc.2026.1917106)
Supplement: Supplementary file 1 [file Table1.docx]

**Supplementary Table S1: List of primers, antibodies, and plasmids used in this study.**

| **List of primers** | | |
| --- | --- | --- |
| **qPCR Primers** | | |
| **Gene** | **Forward** | **Reverse** |
| **47s rRNA** | ACACGCTGTCCTCTGGCGAC | CTAGCCGGGTCACCGGTAG ṁb |
| **SPRTN** | GACAAAGACCGAGAAGGGCA | TAACAGCAGGGGAGACCAGA |
| **ABCB1** | GCTGTCAAGGAAGCCAATGCCT | TGCAATGGCGATCCTCTGCTTC |
| **ABCG2** | GTTCTCAGCAGCTCTTCGGCTT | TCCTCCAGACACACCACGGATA |
| **MRP2** | GCCAACTTGTGGCTGTGATAGG | ATCCAGGACTGCTGTGGGACAT |
| **ZEB1** | CCAGGTGTAAGCGCAGAAAG | TCACTGCTCCTCCCTGGTAA |
| **miR-195-5p** | GTCGTATCCAGTGCAGGGTCCGAGGT | ATTCGCACTGGATACGACTATAACCG |
| **U6** | CTCGCTTCGGCAGCACA | AACGCTTCACGAATTTGCGT |
| **microRNA universal primer** | GGACGGTAGCAAGCAAAGAGTGTG | GGGATTCTGGAAGATGATGATGAC |
| **Twist** | TACGCCTTCTCGGTCTGGAG | CCACGCCCTGTTTCTTTGAA |
| **CDH1** | TGGACAGGGAGGATTTTGAGC | TTGGGTTGGGTCGTTGTACT |
| **BAX** | TCAGGATGCGTCCACCAAGA | TGTGTCCACGGCGGCAATCATC |
| **BCL2** | GTGGTGGAGGAACTCTTCAG | GTTCCACAAAGGCATCCCAG |
| **p21** | GTCACTGTCTTGTACCCTTGTG | CGGCGTTTGGAGTGGTAGAAA |
| **Gamma-Actin** | AGAGCAGACCCTCCCCAC | CCCAGGCCCGTTTGTGAC |
| **TAF1C** | GTGAAGATGCTGGACACTCAGG | ACTGGGTAAGCAGGACACGTTC |
| **ChIP primers** | | |
| **Gene** | **Forward** | **Reverse** |
| **G4-rDNA core promoter** | CAGGTGTCCGTGTCCGTG | GGAGCGAAAGATATACCTCCCC |
| **rDNA core promoter** | GCCCCGGGGGAGGTAT | GAGGACAGCGTGTCAGCAATA |
| **rDNA 5' ETS** | CCGTGCCGAGTCGTGAC | CGCTTTCCCAGGGCCA |
| **SPRTN Promoter** | CCGCGGCTGTCTAGACCCAC | GAAGCTAGTTTCAGCCTTTA |
|  |  |  |
|  |  |  |
| **List of antibodies** | | |
| **Antibody** | **Company** | **Catalogue no.** |
| **Topoisomerase2A** | Affinity Biotech. | AF6729 |
| **SPRTN** | Novus biologicals | NBP2-94506 |
| **NPM-1** | Cloud-Clone Corp. | PAC664Hu01 |
| **TAF1B** | Invitrogen | PA5-104353 |
| **PARP-1** | Novus biologicals | NBP2-13732 |
| **p53** | BioBharathi LifeSciences | BB-AB0100 |
| **GAPDH** | Abcam | ab128915 |
| **Beta-actin** | Cloud-Clone Corp. | PAB340Hu01 |
| **Ki67** | Cell Signalling Technology | 9129 |
| **ZEB1** | Abcam | ab203829 |
| **Rabbit IgG isotype control** | Invitrogen | 10500C |
| **Anti-rabbit IgG, HRP-linked secondary antibody** | Cell Signalling Technology | 7074 |
| **Texas Red secondary antibody** | Merck | SAB3700858 |
| **Alexa Fluor @488 secondary antibody** | Cell-signaling Technology | 4408 |
| **Anti-rabbit IgG (H+L) F (ab')2** | Sigma-Aldrich | SAB4600322 |
|  |  |  |
| **List of Plasmids** | | |
| **Plasmids** | **Company** | **Catalogue no.** |
| **SPRTN_shRNA_plasmid** | Merck | TRCN0000073309 |
| **SPRTN expression vector** | Addgene | 110214 |
| **PARP-1_shRNA_plasmid** | Merck | TRCN0000007929 |
| **ZEB1 expression vector** | Gift from Dr. K.H. Harshan, CCMB, Hyderabad, India | - |
| **Pgl3 control-luc-SPRTN-3' UTR** | Biotech desk | GS-00 |
| **SPRTN CD plasmid** | Gift from Dr. Swagata Halder, Plaksha University | - |
| **TAF1C shRNA plasmid** | Merck | TRCN0000013326 |
